# Effect of the ApoE genotype on the efficacy of multidomain lifestyle interventions on cognitive change: a meta‐analysis of three randomized clinical trials

**DOI:** 10.1002/alz70860_102747

**Published:** 2025-12-23

**Authors:** Jenni Lehtisalo, Alina Solomon, Christelle Cantet, Nicola Coley, Esko Levälahti, Francesca Mangialasche, Tiia Ngandu, Sandrine Andrieu, Hidenori Arai, Miia Kivipelto

**Affiliations:** ^1^ Finnish Institute for Health and Welfare, Helsinki, Finland; ^2^ University of Eastern Finland, Kuopio, Finland; ^3^ Imperial College London, London, United Kingdom; ^4^ Karolinska Institutet, Stockholm, Sweden; ^5^ INSERM‐University of Toulouse, Toulouse, France; ^6^ Toulouse University Hospital, Toulouse, France; ^7^ IHU HealthAge, Toulouse, France; ^8^ Karolinska University Hospital, Stockholm, Sweden; ^9^ FINGERS Brain Health Institute, Stockholm, Sweden; ^10^ IHU HealthAge CERPOP UMR 1295, Toulouse, France; ^11^ National Center for Geriatrics and Gerontology, Obu, Aichi, Japan; ^12^ The Ageing Epidemiology (AGE) Research Unit, School of Public Health, Imperial College London, London, United Kingdom, London, United Kingdom

## Abstract

**Background:**

The apolipoprotein E (ApoE) ε4 allele is the main genetic risk factor for sporadic Alzheimer's Disease and carriers are thus a key target group for prevention. ApoE genotype may also modify the association between lifestyle and cognitive impairment. Benefits of lifestyle‐based preventive interventions have been shown among ApoE4 carriers in stratified analyses, but no individual study or an earlier meta‐analysis has so far shown if benefits are different between carriers and non‐carriers (i.e. statistically significant ApoE*intervention interaction). We conducted a new meta‐analysis with three studies.

**Method:**

Results from the randomized clinical trials FINGER in Finland (*n* = 1259, 60‐77 yrs, at‐risk population based on CAIDE risk score and cognition, 24 months), MAPT in France (*n* = 1679, >=70 yrs, at‐risk population based on subjective complaints or signs of frailty, 36 months) and J‐MINT in Japan (*n* = 531 participants, 65‐85 yrs, MCI population, 18 months) were combined for a meta‐analysis. FINGER and J‐MINT studies randomised participants into multidomain lifestyle intervention (physical activity, healthy diet, cognitive training, risk factor monitoring) and control groups. MAPT combined lifestyle with omega‐3 supplement including 4 arms. The group receiving supplement only was excluded and groups receiving lifestyle intervention with or without supplements were combined. Cognitive change was estimated using the original primary outcome of each trial (composite cognition score based on multiple tests). APOE genotype was dichotomised and carriers of one or two ApoE4 allele were compared with non‐carriers.

**Result:**

Number of participants with ApoE4 information was 1109 in FINGER (362 carriers, 33%), 426 in J‐MINT (124 carriers, 29%), and 934 in MAPT (223 carriers, 24%). Preliminary results show that benefit of the intervention was greater among the ApoE4 carriers compared with non‐carriers (time*group*ApoE4 interaction *p* = 0.035). Effect size was greatest in the J‐MINT (0.36) followed by FINGER (0.15) and MAPT (0.12).

**Conclusion:**

This study provides the first robust evidence that multidomain, lifestyle‐based interventions can particularly benefit ApoE4 carriers. Importantly, ApoE4 was shown to modify the effect of the intervention on cognition across different populations (Asian and European) and cognitive groups (at‐risk and MCI). Thus, ApoE4 carriers represent a particularly important target group for preventive interventions.

